# Regulation of PD‐1PD‐L1 Immune Checkpoints by Gut Microbiota Metabolites and Their Clinical Translational Research: A Review

**DOI:** 10.1002/iid3.70492

**Published:** 2026-07-29

**Authors:** Xuanyou Fang, Shuai Yuan, Weijian Mai

**Affiliations:** ^1^ Xiaolan Clinical Institute of Shantou University Medical College Zhongshan Guangdong Province China

**Keywords:** cancer immunotherapy, clinical translation, gut microbiota, immune checkpoint inhibitors, metabolites, PD‐1, PD‐L1

## Abstract

**Background:**

Immune checkpoint inhibitors (ICIs) targeting the PD‐1/PD‐L1 pathway have revolutionized cancer immunotherapy, yet their clinical benefit is constrained by variable response rates and immune‐related adverse events.

**Aims:**

This review systematically analyzes the molecular mechanisms by which key gut microbiota‐derived metabolites—including short‐chain fatty acids (SCFAs), tryptophan metabolites, and bile acids—modulate the PD‐1/PD‐L1 axis.

**Materials & Methods:**

We synthesized and evaluated peer‐reviewed preclinical and clinical studies published over the past decade, focusing on metabolite–immune interactions, biomarker validation, and combinatorial intervention strategies.

**Results:**

The summarized evidence demonstrates that these metabolites exert significant influences on the tumor microenvironment, enhance T‐cell effector functions, and reshape immune tolerance, thereby affecting ICI responsiveness.

**Discussion:**

We critically assess the predictive value of microbiota metabolites as potential biomarkers and review current progress in probiotic supplementation, fecal microbiota transplantation, and metabolite‐based combination therapies.

**Conclusion:**

Despite promising translational prospects, several challenges—including inter‐individual variability, lack of standardized protocols, and mechanistic gaps—remain to be addressed. Future directions should prioritize large‐scale longitudinal studies and refined intervention designs to facilitate the clinical integration of microbiota‐guided strategies, ultimately improving the precision and efficacy of cancer immunotherapy.

**Abbreviations**
AhR: aryl hydrocarbon receptor
BA: bile acid
BTC: biliary tract cancer
CRC: colorectal cancer
CTL: cytotoxic T lymphocyte
CXCL3: C‐X‐C motif chemokine ligand 3
DAT: desaminotyrosine
DC: dendritic cell
DCA: deoxycholic acid
DCB: durable clinical benefit
FMT: fecal microbiota transplantation
FOXO1: forkhead box protein O1
FXR: farnesoid X receptor
GAPDH: glyceraldehyde‐3‐phosphate dehydrogenase
GCDCA: glycochenodeoxycholic acid
GP: ginseng polysaccharide
GPR109A: G protein‐coupled receptor 109 A
GPR41: G protein‐coupled receptor 41
GPR43: G protein‐coupled receptor 43
H3K27ac: histone 3 lysine 27 acetylation
HCC: hepatocellular carcinoma
HDAC: histone deacetylase
I3C: indole‐3‐carboxaldehyde
ICB: immune checkpoint blockade
ICD: immunogenic cell death
ICI: immune checkpoint inhibitor
IDO: indoleamine 2,3‐dioxygenase
IDO1: indoleamine 2,3‐dioxygenase 1
IFN‐γ: interferon‐gamma
IGF2BP3: insulin‐like growth factor 2 mRNA‐binding protein 3
IL: interleukin
IL‐12: interleukin‐12
IL‐17: interleukin‐17
IL‐1β: interleukin‐1 beta
IL‐22: interleukin‐22
IL‐6: interleukin‐6
iNKT: invariant natural killer T
iNOS: inducible nitric oxide synthase
IPA: indole‐3‐propionic acid
irAE: immune‐related adverse event
JAK: Janus kinase
LCA: lithocholic acid
LC‐MS/MS: liquid chromatography‐tandem mass spectrometry
LSEC: liver sinusoidal endothelial cell
LUAD: lung adenocarcinoma
MDSC: myeloid‐derived suppressor cell
MPR: major pathologic response
MSI: mass spectrometry imaging
NaB: sodium butyrate
NDB: non‐durable benefit
NK: natural killer
NKT: natural killer T
NSCLC: non‐small cell lung cancer
OCA: obeticholic acid
OXPHOS: oxidative phosphorylation
PD‐1: programmed cell death protein 1
PD‐L1: programmed death‐ligand 1
PMN‐MDSC: polymorphonuclear myeloid‐derived suppressor cell
SCFA: short‐chain fatty acid
SP: sodium propionate
TCR: T cell receptor
TGR5: Takeda G protein‐coupled receptor 5
Th1: T helper 1 cell
Th17: T helper 17 cell
TLCA: taurolithocholic acid
TME: tumor microenvironment
TNF‐α: tumor necrosis factor‐alpha
Treg: regulatory T cell
TUDCA: tauroursodeoxycholic acid
TVA: trans‐vaccenic acid
UDCA: ursodeoxycholic acid

## 1 Introduction

The programmed death‐1/programmed death‐ligand 1 (PD‐1/PD‐L1) immune checkpoint pathway is a critical mechanism for maintaining immune homeostasis and preventing autoimmune diseases. However, it also represents a major pathway by which tumors evade immune surveillance [1]. Consequently, inhibitors targeting this axis have become standard‐of‐care for many cancer types, revolutionizing the treatment of advanced malignancies [1]. Nevertheless, the clinical efficacy of ICIs exhibits substantial inter‐individual variability, and a considerable percentage of patients derive no benefit due to primary or acquired resistance [2, 3]. This variability underscores the significant impact of host‐intrinsic factors beyond the tumor itself. A large body of evidence now identifies the gut microbiota—the complex community of commensal microorganisms in the gastrointestinal tract—as a crucial determinant of both the therapeutic efficacy and toxicity profiles of ICIs [4, 5]. Clinical and preclinical studies consistently indicate that the composition and functional state of the gut microbiome can predict patient responses to anti‐PD‐1/PD‐L1 therapy, with specific microbial signatures linked to either durable clinical benefit or treatment failure [6, 7, 8]. In biliary tract cancer (BTC), distinct gut microbial and metabolite profiles distinguished patients with durable clinical benefit from those with non‐durable benefit (NDB) following anti‐PD‐1/PD‐L1 therapy, and predictive models based on these features achieved high accuracy [6]. In extensive‐stage small cell lung cancer, differential enrichment of bacterial taxa such as *Faecalibacterium* and *Clostridium_sensu_stricto_1* in responders after treatment highlights the dynamic role of the microbiota. This close relationship suggests that the gut microbiome is not a passive bystander but an active regulator of systemic antitumor immunity, capable of reshaping the tumor immune microenvironment (TME) and ultimately influencing clinical outcomes [9, 10].

The gut microbiota influences systemic immunity through complex host‐microbe interactions, in which microbial metabolites act as direct and potent signaling molecules [11, 12]. These small molecules, derived from bacterial fermentation of dietary components or from modification of host‐derived compounds, enter the circulation and orchestrate antitumor immune responses locally or systemically. The regulation of the PD‐1/PD‐L1 pathway is a key aspect of this microbial‐host crosstalk [13]. Various metabolites—including SCFAs, tryptophan catabolites, bile acids, and others—have been implicated in fine‐tuning the expression and function of PD‐1 and PD‐L1 on tumor and immune cells, thereby shifting the balance between immune activation and tolerance [14, 15, 16]. For example, butyrate, an SCFA produced by bacteria such as *Clostridia* and *Lachnospiraceae*, enhances anti‐PD‐1 therapy efficacy by modulating T‐cell receptor signaling and improving the stemness and functionality of cytotoxic CD8^+^ T cells [17, 18]. Conversely, microbial metabolites can also foster an immunosuppressive environment. High‐dose sodium propionate (SP) increases PD‐L1 expression in colorectal cancer (CRC) cells via the IGF2BP3 axis, potentially aiding immune escape [19]. Additionally, tryptophan breakdown by the host enzyme indoleamine 2,3‐dioxygenase 1 (IDO1) or by gut microbes produces kynurenine, which activates the aryl hydrocarbon receptor (AhR) and can upregulate PD‐L1 expression and promote T‐cell exhaustion, illustrating the interconnection between microbial metabolism, the AhR pathway, and PD‐1/PD‐L1 checkpoint regulation [20, 21]. This complex metabolic interplay establishes gut microbiota‐derived metabolites as master regulators that can either enhance or blunt PD‐1/PD‐L1 blockade, making them attractive targets for therapeutic intervention [22].

This review aims to provide a comprehensive, mechanistic synthesis of the precise regulatory roles of various classes of gut microbial metabolites on the PD‐1/PD‐L1 immune checkpoint axis. We examine the molecular mechanisms by which metabolites such as SCFAs (e.g., butyrate, propionate, acetate), tryptophan derivatives (e.g., kynurenine, indole compounds), bile acids (e.g., secondary bile acids, tauroursodeoxycholic acid), and other important molecules (e.g., inosine, desaminotyrosine) regulate PD‐1 and PD‐L1 expression and signaling on T cells, dendritic cells (DCs), macrophages, and tumor cells [14, 23]. The discussion also covers how these metabolites reshape the overall immune landscape of the tumor, affecting the infiltration and function of different immune cell types. The review then critically assesses the translational potential of these findings, evaluating current strategies and evidence for converting microbiome and metabolite insights into clinical tools that enhance cancer immunotherapy outcomes. This includes an analysis of microbiota‐targeted interventions—fecal microbiota transplantation (FMT), probiotics, prebiotics, postbiotics, dietary modifications (e.g., ketogenic diets, fiber supplementation), and engineered microbial therapies—that have shown promise in preclinical models and early clinical trials for increasing tumor sensitivity to anti‐PD‐1/PD‐L1 therapy [24, 25, 26, 27]. We also discuss the challenges and future opportunities in this rapidly evolving field, such as the need for standardized interventions, understanding context‐dependent effects of metabolites, and integrating multi‐omics approaches for patient stratification and personalized, microbiome‐guided immunotherapy regimens [28, 29]. A comprehensive summary of gut microbiota‐derived metabolites, their mechanisms, context‐dependent effects, and clinical potential is provided in Table 1.

**Table 1:** Gut microbiota‐derived metabolites modulating the PD‐1/PD‐L1 axis.

Metabolite class	Specific metabolite	Mechanism and immune effects	Condition‐dependent effects	Impact on PD‐1/PD‐L1 axis	Clinical potential
Short‐chain fatty acids	Butyrate	HDAC inhibition; enhances T cell stemness (FOXO1); improves TCR signaling	*Concentration*: enhances anti‐PD‐1 efficacy at physiological levels; *Tumor type*: upregulates PD‐L1 in glioma (PI3K/AKT), downregulates in CRC (STAT1 degradation); *Microenvironment*: modulated by gut microbiota composition	Enhances CD8^+^ T cell function; upregulates PD‐1; modulates PD‐L1 in tumor‐specific manner	Improves ICIs efficacy; mitigates ICIs‐related cardiotoxicity; biomarker
	Propionate	GPR43 agonist; promotes oxidative phosphorylation; inhibits STAT1/STAT3	*Concentration*: low dose enhances antitumor immunity; high dose may upregulate PD‐L1 in CRC	Reduces PD‐1 expression; enhances memory T cell phenotype	Enhances ICIs efficacy; requires dose optimization
	Acetate	Increases acetyl‐CoA; enhances GAPDH acetylation; promotes c‐Myc acetylation	*Tumor type*: upregulates PD‐L1 on tumor cells via c‐Myc acetylation	Promotes Th1 differentiation; upregulates PD‐L1 on tumor cells	Potential ICIs combination; biomarker for immune evasion
Tryptophan metabolites	Indole‐3‐propionic acid	AhR agonist; promotes T cell stemness	*Consistent immunostimulatory* across pan‐cancer models	Enhances ICIs efficacy	ICIs combination therapy
	Indole‐3‐carboxaldehyde	AhR agonist; enhances IL‐22 pathway; maintains intestinal barrier	*Microenvironment*: protects against ICI‐induced colitis without compromising antitumor efficacy	Reduces ICIs‐related toxicity	Reduces ICIs toxicity; preserves antitumor immunity
	Kynurenine	AhR agonist; induces chromatin remodeling; promotes Treg expansion	*Consistent immunosuppressive* across multiple tumor types; correlates with ICIs resistance	Upregulates PD‐1; induces T cell exhaustion	Biomarker for ICIs resistance; therapeutic target
Bile acids	Lithocholic acid derivatives	RORγt inhibitor; regulates Th17/Treg balance	*Concentration*: promotes Treg differentiation at low levels; inhibits Th17 at high levels	Indirectly modulates immune checkpoint via T cell subsets	Improves immune balance; biomarker for ICIs response
	Deoxycholic acid	Activates TGR5/STAT3 signaling	*Tumor type*: promotes PD‐L1 upregulation and immune evasion in lung cancer	Upregulates PD‐L1; promotes immune evasion	Biomarker for ICIs response; therapeutic target
	Ursodeoxycholic acid	Not fully defined	*Favorable context*: enriched in ICIs responders; associated with *Lachnoclostridium*	Associated with favorable ICIs response	Biomarker for ICIs response
Other metabolites	Inosine	Enhances T cell function	*Consistent immunostimulatory* across models	Enhances anti‐PD‐1 efficacy	ICIs combination therapy
	4‐Hydroxyphenylacetic acid	Activates JAK2/STAT3; induces CXCL3 expression	*Pro‐tumor context*: promotes PMN‐MDSC recruitment and CD8^+^ T cell suppression in CRC	Promotes immunosuppressive microenvironment	Therapeutic target
	Phenylacetylglutamine	Not fully defined	*Negative context*: associated with ICIs resistance across pan‐cancer cohorts	Negatively associated with ICIs efficacy	Biomarker for ICIs resistance

*Data sources:* Butyrate [17, 30, 31]; propionate [32, 33]; acetate [34, 35]; indole‐3‐propionic acid [36]; indole‐3‐carboxaldehyde [37]; kynurenine [38, 39, 40, 41, 42]; lithocholic acid derivatives [43]; deoxycholic acid [44]; ursodeoxycholic acid [45]; inosine [46]; 4‐hydroxyphenylacetic acid [47]; phenylacetylglutamine [48]. Additional mechanistic insights from reviews [15, 28].

## 2 Overview of the Interaction Between Gut Microbiota Metabolites and the Immune System

### 2.1 Classification and Sources of Major Immunomodulatory Metabolites

The gut microbiota produces a wide array of metabolites that critically modulate the host immune system. These metabolites can be grouped into several main classes based on their chemical structure and biosynthetic pathways. Specific bacteria, mostly from the phyla Bacteroidetes and Firmicutes, break down dietary fibers into SCFAs, including acetate, propionate, and butyrate [49]. These metabolites are important energy sources for colonocytes and also act as histone deacetylase (HDAC) inhibitors and ligands for G‐protein‐coupled receptors (GPCRs), thereby affecting immune cell development and inflammatory responses [50]. Another major group of immunomodulatory compounds comprises tryptophan metabolites. Gut bacteria, including some *Lactobacillus* species, metabolize dietary tryptophan via the kynurenine pathway and an IDO‐independent pathway. The latter produces indoles such as indole‐3‐propionate and indole‐3‐acetate, which are important for maintaining gut barrier integrity and regulating the balance between T helper 17 (Th17) cells and regulatory T cells (Tregs) [51]. Bile acids represent the third important class extensively modified by the gut microbiota. Bacteria such as *Bacteroides* and *Clostridia* dehydroxylate primary bile acids from the liver to generate secondary bile acids, including deoxycholic acid (DCA) and lithocholic acid (LCA) [52]. These secondary bile acids bind to nuclear receptors such as the farnesoid X receptor (FXR) and the membrane receptor Takeda G‐protein‐coupled receptor 5 (TGR5), playing important roles in lipid metabolism, inflammation, and immune homeostasis [53]. Various gut microbes also produce polyamines (e.g., putrescine, spermidine), which are essential for basic cellular processes such as growth and differentiation, and modulate T‐cell and macrophage function, thereby influencing overall immunity [54]. The production and relative abundance of these metabolites are strongly influenced by the composition of the gut microbiota, which is itself shaped by host genetics, diet, and environmental factors [55]. Therefore, a detailed understanding of the classification and microbial origins of these immunomodulatory metabolites is essential for elucidating their roles in health and disease and for developing targeted therapeutic strategies.

### 2.2 General Mechanisms by Which Metabolites Influence Immunity

Gut microbiota‐derived metabolites exert broad effects on host immunity through several key, interconnected mechanisms. One major mechanism is epigenetic regulation. Metabolites such as the SCFA butyrate act as HDAC inhibitors, altering chromatin accessibility at promoters of immune‐related genes and thereby affecting immune cell development and function. For example, butyrate promotes colonic Treg differentiation by increasing expression of the transcription factor Foxp3, a master regulator of Treg development [56]. Conversely, butyrate can inhibit the production of pro‐inflammatory cytokines such as interferon‐gamma (IFN‐γ) and interleukin‐17 (IL‐17) in effector T cells, thus promoting immune homeostasis [57]. This epigenetic regulation extends beyond T cells: SCFAs also suppress the activation of innate immune cells, including monocytes, myeloid DCs, and plasmacytoid DCs, reducing levels of pro‐inflammatory cytokines such as tumor necrosis factor‐alpha (TNF‐α), IL‐6, and IL‐1β [57]. The ability of metabolites like butyrate and propionate to induce innate immune tolerance further underscores their immunomodulatory functions [57]. A second important mechanism is receptor‐mediated signal transduction. Metabolites bind to specific receptors on immune and epithelial cells. Key receptors include GPCRs such as GPR41, GPR43, and GPR109A, which are activated by SCFAs [32, 58]. Activation of these receptors initiates downstream signaling cascades (e.g., the cAMP‐PKA‐CREB axis) that can enhance CD8^+^ T‐cell function, as exemplified by trans‐vaccenic acid (TVA) acting as a GPR43 antagonist [59]. The AhR is another critical receptor activated by tryptophan metabolites such as indole‐3‐carboxaldehyde (I3C), kynurenine, and other indole derivatives [60, 61]. AhR activation is essential for maintaining epithelial barrier integrity, increasing IL‐22 production, and regulating T‐cell differentiation and immune homeostasis [37, 62]. Furthermore, bile acid receptors such as FXR and TGR5 are activated by primary and secondary bile acids, influencing immune cell functions including Treg differentiation and suppression of pro‐inflammatory cytokine production in conditions such as myasthenia gravis and ulcerative colitis [63, 64, 65]. Third, metabolites directly participate in cellular metabolic reprogramming. They serve as substrates or signaling molecules that affect the bioenergetic and biosynthetic pathways of immune cells, thereby dictating their functional fate. For instance, acetate promotes T helper 1 (Th1) cell differentiation by elevating acetyl‐coenzyme A (acetyl‐CoA) levels and enhancing the acetylation and activity of glyceraldehyde‐3‐phosphate dehydrogenase (GAPDH), thus increasing glycolytic flux [34]. In contrast, propionate inhibits pro‐inflammatory signaling in CD4^+^ T cells by blocking STAT1 and STAT3 phosphorylation, which are critical for Th1 and Th17 responses, respectively; this effect depends on free fatty acid receptor signaling [32]. Microbial metabolites such as inosine and desaminotyrosine (DAT) can augment antitumor T‐cell priming and function, with DAT's effect requiring host type I interferon signaling [23, 46]. Sodium butyrate (NaB) enhances the antitumor efficacy of chimeric antigen receptor (CAR) T cells by simultaneously activating glycolytic and oxidative phosphorylation pathways and upregulating genes involved in extracellular matrix remodeling [66]. Finally, metabolites play a crucial role in maintaining intestinal barrier integrity and modulating systemic immunity. SCFAs and tryptophan catabolites strengthen the gut epithelial barrier by increasing mucus production and promoting the expression of tight junction proteins (e.g., claudin‐1, occludin) [62, 67]. This reduces bacterial translocation and subsequent inflammation, creating a more favorable systemic immune environment and, in the context of cancer, may potentiate antitumor immunity [28, 68]. The integrity of this barrier is vital for preventing leakage of microbial products that could induce chronic, low‐grade inflammation, which is detrimental to immune homeostasis and may compromise responses to therapies, including ICIs [37]. In summary, gut microbiota‐derived metabolites are essential communicators that fine‐tune both local and systemic immune responses through epigenetic regulation, receptor signaling, metabolic reprogramming, and barrier maintenance. They influence susceptibility to a wide range of diseases, including infections, autoimmune disorders, and cancer.

## 3 Regulatory Mechanisms of SCFAs on the PD‐1/PD‐L1 Pathway

### 3.1 Enhancing CD8^+^ T Cell Function and Inhibiting PD‐1 Expression

The SCFA butyrate, a major gut microbial metabolite, plays a pivotal role in enhancing the effector function of cytotoxic CD8^+^ T cells and modulating PD‐1 expression through epigenetic mechanisms. Butyrate acts as an HDAC inhibitor, leading to increased histone acetylation at key gene promoters. Specifically, it increases histone 3 lysine 27 acetylation (H3K27ac) at the promoter region of the *Pdcd1* gene (encoding PD‐1), thereby promoting PD‐1 expression in human CD8^+^ T cells [17]. Paradoxically, while this epigenetic upregulation of PD‐1 occurs, butyrate simultaneously enhances the antitumor efficacy of anti‐PD‐1 therapy by modulating T‐cell receptor (TCR) signaling in cytotoxic CD8^+^ T cells, boosting their production of antitumor cytokines such as IFN‐γ [17]. This suggests a complex regulatory role in which butyrate may prime T cells for a more effective response upon checkpoint blockade. Furthermore, butyrate promotes a stem‐like phenotype in tumor‐specific CD8^+^ T cells within tumor‐draining lymph nodes by inducing a FOXO1‐driven transcriptional program, which is crucial for preserving long‐term T‐cell immunity and is associated with improved responses to immune checkpoint blockade (ICB) in melanoma models [30]. This stemness program is essential for generating durable effector cells. In addition to its direct effects on T cells, butyrate enhances CD8^+^ T‐cell responses by promoting the IL‐12 signaling pathway in an ID2‐dependent manner, further linking microbial metabolites to cytotoxic T‐cell function [31]. The net effect of butyrate is to expand the pool and functionality of tumor‐specific CD8^+^ T cells, making them more responsive to immunotherapy, even as it epigenetically influences checkpoint molecule expression.

Propionate, another important SCFA, sustains CD8^+^ T‐cell fitness by altering cellular energy metabolism. Propionate signals through GPR43, a pathway that regulates immune cells. Propionate activates GPR43, which can shift CD8^+^ T cells toward oxidative phosphorylation (OXPHOS), a metabolic state associated with improved memory formation and longevity [33]. This metabolic shift away from glycolysis and toward OXPHOS is characteristic of long‐lived, stem‐like, and memory T‐cell subsets. Propionate promotes this metabolic phenotype, thereby helping CD8^+^ T cells survive in the harsh tumor microenvironment. Simultaneously, exposure to propionate correlates with reduced surface expression of the inhibitory receptor PD‐1 on CD8^+^ T cells [33]. Lower PD‐1 levels make these T cells less susceptible to inhibition by PD‐L1 on tumor or stromal cells, potentially enhancing their cancer‐killing ability. Thus, propionate is an important metabolite for maintaining an effective antitumor T‐cell response by promoting a metabolically robust, memory‐prone state and reducing exhaustion markers such as PD‐1. The role of SCFAs in altering T‐cell metabolism and exhaustion markers indicates a direct microbial metabolite‐mediated pathway to improve T‐cell function in cancer immunotherapy.

SCFAs also have potent indirect effects on CD8^+^ T‐cell priming and function by modulating antigen‐presenting cells, particularly DCs. SCFAs such as butyrate and propionate influence DC maturation and functional polarization, enhancing the capacity of DCs to produce pro‐inflammatory and immunostimulatory cytokines, including IL‐12 [69]. IL‐12 is a critical cytokine that promotes the differentiation of naïve T cells into Th1 cells and strongly boosts the activation and effector functions of CD8^+^ T cells, such as IFN‐γ production. By making DCs more immunogenic, SCFAs create a lymphoid microenvironment that encourages the generation of robust effector T cells rather than tolerogenic or regulatory responses. This enhanced DC function facilitates strong priming of naïve CD8^+^ T cells, helping them become cytotoxic effectors that can better target tumors. Moreover, the cytokine environment shaped by SCFA‐educated DCs can impair the differentiation or function of immunosuppressive cell types such as Tregs within the tumor‐draining lymph nodes and the TME [69]. Consequently, the CD8^+^ T cells generated under this influence are not only more numerous and functionally active but also operate in an environment with reduced immunosuppressive pressure. This indirect, DC‐mediated pathway amplifies systemic antitumor immunity and renders the effector T‐cell pool less sensitive to inhibitory signals such as those transmitted through PD‐1, synergizing with the direct T‐cell metabolic and epigenetic reprogramming induced by SCFAs.

### 3.2 Modulating Myeloid‐Derived Suppressor Cells and Tumor Cell PD‐L1 Expression

SCFAs such as butyrate and propionate also play significant roles in modulating the immunosuppressive TME, particularly by targeting myeloid‐derived suppressor cells (MDSCs). MDSCs are key drivers of immune evasion, partly by facilitating the PD‐1/PD‐L1 axis. Research indicates that SCFAs can inhibit the accumulation and immunosuppressive function of MDSCs. For instance, a review on lung cancer immunotherapy highlights that microbial metabolites, including SCFAs, influence MDSCs, which are central to creating an immunosuppressive milieu that contributes to T‐cell exhaustion and limits the efficacy of PD‐1/PD‐L1 inhibitors [70]. By modulating these cells, SCFAs can reduce the secretion of immunosuppressive factors such as arginase‐1 (ARG1) and inducible nitric oxide synthase (iNOS). This effect is supported by a study on decursin, an anti‐cancer compound, which showed that treatment attenuated MDSC‐mediated immune suppression, as indicated by reduced Arg1 expression, and concurrently improved the accumulation of cytotoxic T cells in the TME [71]. This reduction in MDSC activity helps relieve T‐cell inhibition, thereby indirectly weakening PD‐1/PD‐L1‐mediated immune escape. The interplay between diet, microbiota, and MDSCs is further illustrated in a preclinical colon cancer model investigating dietary fiber. That study found that cured mice following combined radio‐immunotherapy exhibited decreased proportions of MDSCs in the spleen alongside increased CD8^+^ T‐cell proportions, suggesting that dietary components influencing the microbiome can alter MDSC levels and improve therapeutic outcomes, potentially impacting PD‐1/PD‐L1 checkpoint efficacy [72].

**Figure 1:**
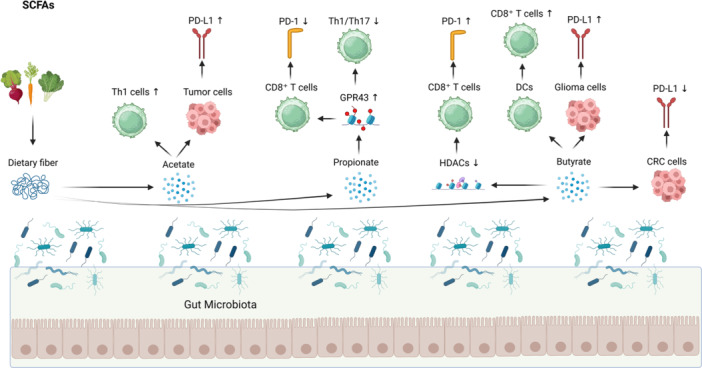
Short‐chain fatty acids (SCFAs) pathway modulating the PD‐1/PD‐L1 Axis.

Emerging evidence also suggests that SCFAs may directly or indirectly affect tumor cell PD‐L1 expression through intracellular metabolic and signaling pathways, although the precise mechanisms require further investigation across different cancer types. The hypoxic TME, regulated by factors such as HIF‐1α, is known to induce PD‐L1 expression. A study on decursin showed that it promotes HIF‐1α proteasomal degradation under hypoxic conditions, thereby reducing PD‐L1 expression in an allograft mouse tumor model [71]. This link between hypoxia, metabolism, and immune checkpoint expression suggests a possible indirect route by which microbial metabolites or their downstream effects could alter PD‐L1. Additionally, altering the energy metabolism of tumor cells, especially in therapy‐resistant settings, is closely linked to PD‐L1 regulation. A study on platinum‐resistant non‐small cell lung cancer (NSCLC) found that resistant tumors have a more immunosuppressive environment and higher PD‐L1 expression [73]. Although that study focused on kynurenine pathway enzymes IDO1/TDO2 rather than SCFAs, it demonstrates that changes in tumor cell metabolism, which could be influenced by microbial metabolites, can directly affect PD‐L1 levels and the overall immune landscape. Blocking those metabolic pathways boosted antitumor immunity and synergized with PD‐1 blockade, indicating that metabolite‐based strategies could lower PD‐L1 levels [73]. Although direct evidence linking SCFAs to PD‐L1 downregulation in tumor cells is lacking and likely cancer‐type specific, current data on metabolic remodeling of the TME strongly support this notion. The integration of microbiome science into lung cancer immunotherapy points to a future where understanding these metabolic interactions will be crucial for developing strategies to target both MDSCs and tumor cell PD‐L1 to overcome ICI resistance [70]. In summary, SCFAs modulate antitumor immunity through multiple pathways, as illustrated in Figure 1.

## 4 Tryptophan Metabolites Regulate Immune Checkpoints via the AhR

### 4.1 Direct Effects of AhR Ligands on PD‐1 Expression

Indole‐3‐carboxaldehyde (I3C) and indole‐3‐propionic acid (IPA) are indole metabolites produced by the gut microbiota that bind to the AhR, a ligand‐activated transcription factor highly expressed on many immune cells, including T cells, DCs, and Tregs [74, 75]. These ligands activate AhR signaling, which plays an important and context‐dependent role in modulating immune responses, especially within the TME. AhR activation is often associated with immunosuppression in cancer. In lung adenocarcinoma (LUAD), AhR mediates IFN‐γ‐induced immunosuppression by regulating the upregulation of immune checkpoint molecules such as PD‐L1 and IDO1 through the Janus kinase (JAK)/STAT pathway [38, 39]. This pathway creates a feedback loop in which AhR‐driven IDO1 synthesizes kynurenine, another potent AhR ligand that amplifies immunosuppressive signaling [38]. When AhR is activated in the TME, it generally promotes Treg differentiation and function while increasing PD‐1 expression on CD8^+^ T cells and Tregs, thereby fostering immune tolerance and exhaustion [40, 46]. This is evident in models where ligands such as 3,4‐DAA activate AhR and increase the numbers of Tregs and PD‐1^+^ T cells, promoting immune tolerance and reducing liver transplant rejection [76]. In ovarian cancer, the tryptophan metabolite kynurenine, generated by tumor‐expressed IDO1, activates AhR in T cells, resulting in elevated PD‐1 expression through chromatin accessibility changes in the PD‐1 promoter region. This AhR‐mediated axis also operates in other cancers, such as oral squamous cell carcinoma (OSCC) and CRC, reducing antitumor immunity and regulating multiple immune checkpoints [77, 78].

However, the immunological effects of AhR signaling are complex and appear contradictory depending on the ligand, cell type, and disease model. Although AhR activation generally suppresses immunity in cancer, it can sometimes provoke pro‐inflammatory responses, such as Th17 cell differentiation, or exert anti‐inflammatory effects [75]. In autoimmune neuroinflammatory models, such as multiple sclerosis, astrocytic AhR activation in response to inflammatory signals upregulates PD‐L1, which then interacts with PD‐1 on microglia to reduce CNS inflammation, illustrating a protective, anti‐inflammatory function [79]. This duality is also evident in non‐cancer inflammatory settings. Researchers have identified a protective subset of invariant natural killer T (iNKT) cells expressing PD‐L1 in models of airway inflammation induced by fine particulate matter (PM2.5); these cells prevent pathogenic γδ T cells from functioning, at least partly through PD‐1/PD‐L1 signaling, demonstrating a scenario where this axis limits inflammation [80]. In allergic asthma models, PD‐1 acts as a metabolic checkpoint on type‐2 innate lymphoid cells (ILC2s), and PD‐1 agonism ameliorates airway hyperreactivity, indicating an inhibitory regulatory function in a non‐malignant, type‐2 immune setting [81, 82]. Research on urolithins—gut metabolites from pomegranate polyphenols that act on AhR—has shown that AhR effects are ligand‐specific. In glioblastoma, urolithin A and B prevent TNF‐α‐induced PD‐L1 expression, and AhR blockade reduces PD‐L1, suggesting that inhibiting AhR can reverse immunosuppressive signals [83]. Moreover, baseline AhR expression levels significantly impact immune responses to AhR‐modulating drugs. For instance, in pancreatic cancer, the effects of AhR agonists and antagonists on PD‐1/PD‐L1 levels in peripheral blood mononuclear cells differed markedly between patients with high/medium versus low AhR expression [84]. The complex and sometimes opposing regulation of PD‐1 by the AhR pathway, depending on the delicate balance of ligands and microenvironmental signals, highlights its potential as a therapeutic target. Researchers are currently investigating AhR blockade to boost immunity in cancer patients or modulation with specific agonists in autoimmune or allergic diseases as ways to manipulate immune checkpoint regulation for therapeutic benefit [40, 75, 85].

### 4.2 Synergistic Effects of Regulating the IDO Pathway With PD‐1/PD‐L1

The IDO pathway, particularly IDO1, is an important immunosuppressive pathway that synergizes with the PD‐1/PD‐L1 checkpoint to facilitate tumor immune evasion. Tumor cells and DCs often highly express IDO1, which converts tryptophan (Trp) into kynurenine [86]. This metabolic change has two immunosuppressive consequences: it depletes local tryptophan, which inhibits T‐cell growth and function, and it produces kynurenine, a potent AhR ligand [87]. Kynurenine binding to AhR initiates a transcriptional program that increases PD‐1 expression on cytotoxic T lymphocytes (CTLs) and promotes Treg expansion, creating a self‐reinforcing immunosuppressive loop [42]. Because of this complex crosstalk, PD‐1/PD‐L1 blockade may be less effective in tumors with an active IDO‐AhR axis, as the pathway continuously delivers signals that induce T‐cell exhaustion and tolerance. Consequently, disrupting this metabolic‐immune circuit is a logical strategy to enhance ICI therapy. The gut microbiota and its metabolites are emerging as important players in this axis. Microbial metabolites can affect this loop either by interfering with AhR activation or by directly altering IDO1 expression. For example, some indole derivatives produced by gut microbiota from tryptophan metabolism can themselves act as AhR ligands, potentially competing with kynurenine and modulating downstream immunosuppressive signaling [15]. Additionally, the overall composition of the gut microbiota influences systemic immune tone and inflammation, which in turn regulates IDO1 expression in both the TME and peripheral tissues [88]. Thus, microbial metabolites constitute a natural reservoir of compounds capable of disrupting the IDO‐AhR‐PD‐1 axis, offering an innovative approach to dismantle this immunosuppressive network and increase tumor sensitivity to anti‐PD‐1/PD‐L1 therapy.

**Figure 2:**
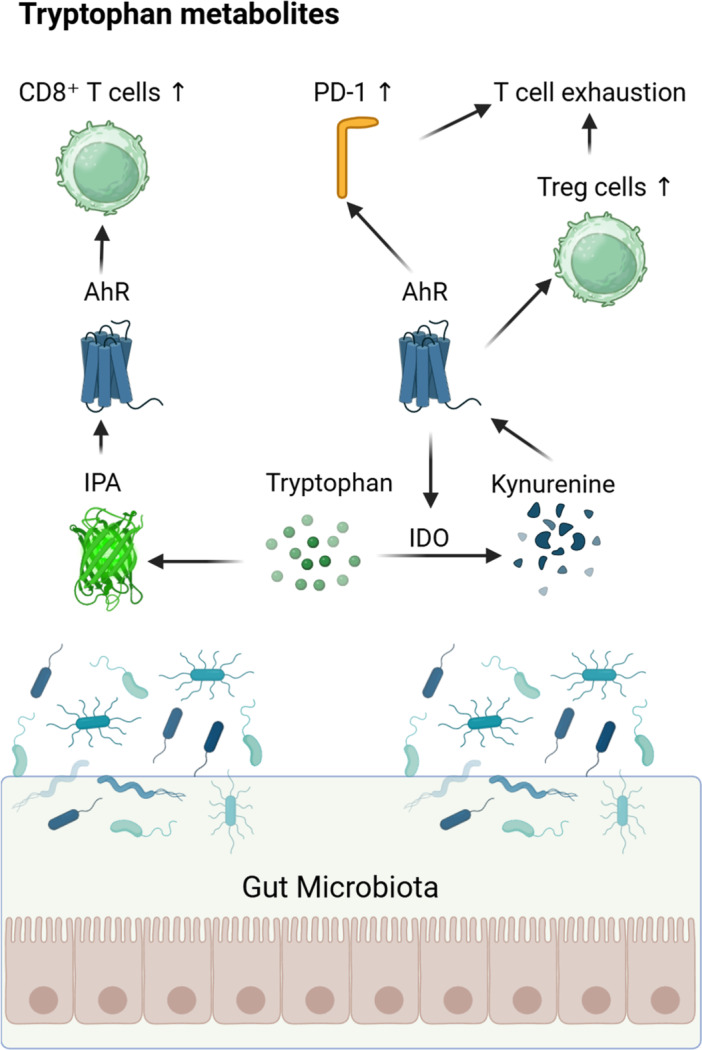
Tryptophan metabolite–AhR pathway modulating the PD‐1/PD‐L1 Axis.

Preclinical and emerging clinical evidence strongly supports the therapeutic potential of targeting the IDO pathway to enhance anti‐PD‐1/PD‐L1 blockade, with microbiota‐derived or microbiota‐influenced inhibitors showing promise. Natural compounds and pharmacological inhibitors that suppress IDO activity reduce kynurenine production, thereby decreasing AhR activation and its downstream effects on PD‐1 upregulation and Treg expansion [89]. For instance, the natural flavonoid icariside I, which can be produced by microbes, significantly lowers kynurenine levels and key enzymes in the kynurenine‐AhR pathway in tumor cells and mouse models [42]. This inhibition reduces nuclear PD‐1 in CTLs and markedly increases tumor‐infiltrating CD8^+^ T cells, ultimately enhancing antitumor immunity [42]. Likewise, ginseng polysaccharides (GPs) modify the gut microbiota, resulting in reduced plasma l‐kynurenine and a lower Kyn/Trp ratio. This alteration facilitates Treg suppression and effector T‐cell activation, thereby enhancing the antitumor efficacy of anti‐PD‐1 monoclonal antibody therapy [88]. Extensive research is ongoing on direct IDO1 inhibitors such as 1‐methyl‐tryptophan (1‐MT) and NLG919. When administered simultaneously with chemotherapy drugs or as part of photodynamic therapy via nanoplatforms, NLG919 stops tryptophan metabolism, reverses the immunosuppressive TME, and boosts CD8^+^ T‐cell activity, thereby improving immunotherapy effectiveness [90, 91]. This combination works even on “immune‐cold” tumors such as osteosarcoma and pancreatic cancer, where IDO‐mediated immunosuppression is a major obstacle [90, 92]. Clinically, elevated IDO activity, indicated by an increased Kyn/Trp ratio, is consistently associated with primary resistance to ICIs in cancers such as NSCLC, highlighting its significance as a predictive biomarker and a potent therapeutic target [93, 94]. Therefore, strategies using microbiota‐modulating agents or direct IDO inhibitors to block the kynurenine‐AhR pathway hold considerable potential for disrupting immune tolerance and achieving synergistic efficacy with PD‐1/PD‐L1 checkpoint blockade. The crosstalk between tryptophan metabolites and the AhR pathway, and its impact on PD‐1/PD‐L1 expression, is depicted in Figure 2.

## 5 Regulation of the PD‐1/PD‐L1 Pathway by Bile Acids Metabolites

### 5.1 Enhancing Anti‐Tumor Immunity Through FXR and TGR5 Receptors

**Figure 3:**
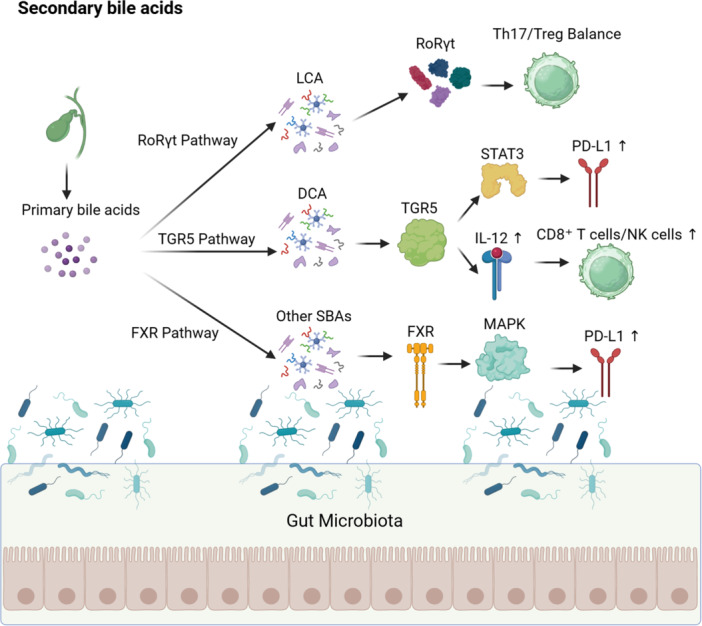
Bile acid metabolic pathway modulating the PD‐1/PD‐L1 axis.

Secondary bile acids derived from the gut microbiota are important signaling molecules that modulate host immunity, including antitumor immunity, through specific receptors such as FXR and the G protein‐coupled bile acid receptor 1 (TGR5, also known as GPBAR1) [95]. Certain secondary bile acids, such as LCA derivatives, have been found to control the differentiation of T helper cells. For example, 3‐oxoLCA and isoalloLCA can promote the differentiation of Th17 cells and Tregs, thereby shifting the balance between pro‐inflammatory and tolerogenic immune states [43]. Notably, some secondary bile acids can inhibit RORγt, the master regulator of Th17 cell development, leading to a reduction in pathogenic IL‐17‐producing Th17 cells [43]. By altering T‐cell subsets, microbial metabolites can reshape the immune landscape, affecting tumor growth and responses to therapies such as ICIs. Bile acids activate FXR, which has multiple effects on the TME. FXR is expressed in many tissues and immune cells, not only in the liver and intestine, the primary bile acid‐targeted organs; for example, it is also found in the lung [96]. Dysregulation of FXR can contribute to various diseases, including cancer [97]. In hepatocellular carcinoma (HCC), the bile acid norcholic acid (NorCA) promotes tumor growth and immune escape by blocking FXR signaling [98]. FXR blockade increases PD‐L1 expression on HCC cells and their exosomes, dampening immune activity and reducing CD4^+^ T‐cell function [98]. Conversely, activating FXR can help fight tumors. In CRC, the FXR agonist GW4064 elicited immunogenic cell death (ICD) in tumor cells [99]. However, an intriguing observation was that GW4064 also increased PD‐L1 expression in CRC cells via FXR and MAPK signaling, suggesting that FXR activation may both boost antitumor immunity and trigger a compensatory immune resistance mechanism [99]. This duality underscores the importance of context and combinatorial strategies. Moreover, FXR activation affects the TME beyond direct effects on tumor cells; it can inhibit the activation of cancer‐associated fibroblasts (CAFs), potentially improving the TME and modulating immune checkpoint molecule expression [97]. In a study of liver cancer, nanoparticles delivering the FXR agonist obeticholic acid (OCA) directly to liver sinusoidal endothelial cells (LSECs) increased CXCL16 secretion, leading to the recruitment and activation of natural killer T (NKT) cells and effective antitumor immunotherapy [100]. This demonstrates that precisely manipulating FXR signaling in specific compartments of the TME can boost intrinsic antitumor defenses. Along with FXR, bile acids also activate TGR5, another powerful immunomodulatory pathway. TGR5 activation in DCs induces IL‐12, a key cytokine that promotes the activation and cytotoxicity of CD8^+^ T cells and natural killer (NK) cells [95]. This enhancement of effector cell function directly increases their tumor‐killing capacity and may reduce their reliance on PD‐1/PD‐L1 checkpoint inhibition. Evidence from lung cancer models corroborates this mechanism. The traditional Chinese medicine formula Yi‐Fei‐San‐Jie (YFSJF) was found to exert antitumor effects by altering bile acid metabolism, specifically by inhibiting DCA breakdown [44]. This metabolic change affected immune regulation by acting on TGR5, suppressing the TGR5/STAT3/PD‐L1 signaling axis [44]. Blocking this pathway led to lower PD‐L1 levels, reduced PD‐1/PD‐L1 interaction, and increased T‐cell immune activity, effectively preventing immune escape in lung cancer [44]. This reveals a direct connection between bile acid metabolism, TGR5 signaling, and the PD‐1/PD‐L1 checkpoint. The interplay between FXR and TGR5 signaling in shaping antitumor immunity holds promise for clinical translation. Combining FXR agonists with PD‐1/PD‐L1 inhibitors has shown synergistic potential. In HCC, the FXR agonist GW4064 synergized with anti‐PD‐1 antibody to inhibit tumor growth, counteracting NorCA‐induced immunosuppression [98]. In CRC, the combination of GW4064 and a PD‐L1 antibody exhibited strong antitumor effects, increasing CD8^+^ T‐cell infiltration and even curing some tumor‐bearing mice, whereas GW4064 alone was ineffective in vivo [99]. These results indicate that modulating bile acid receptors can enhance antitumor immunity but may also upregulate compensatory immune checkpoints such as PD‐L1, making combination therapy with ICIs a rational and effective strategy for achieving durable antitumor responses (Figure 3).

### 5.2 Bile Acids Profiles as Biomarkers for ICIs Efficacy

Increasing clinical evidence indicates that specific bile acid profiles, particularly in feces and plasma, can serve as non‐invasive biomarkers to predict ICI efficacy. A groundbreaking discovery in melanoma patients showed that those who responded to PD‐1 blockade had higher levels of certain secondary bile acids in their feces, which were linked to an increase in beneficial gut bacteria and better clinical outcomes [101]. This association extends beyond melanoma. In patients with unresectable HCC who had an objective response to ICIs, distinct fecal microbial and bile acid signatures were observed, including higher abundances of ursodeoxycholic acid (UDCA) and ursocholic acid, metabolites strongly correlated with the abundance of *Lachnoclostridium* [45]. The combination of *Lachnoclostridium* enrichment and *Prevotella 9* depletion created a microbial signature that strongly predicted better overall survival and was validated in an independent cohort [45]. Similarly, a comprehensive metabolomic analysis of NSCLC identified specific bile acids associated with treatment efficacy. Glycochenodeoxycholic acid (GCDCA) and taurolithocholic acid (TLCA) were linked to better survival and response to immunotherapy; notably, TLCA improved T‐cell activation and antitumor immunity in preclinical models [102]. In addition, a prognostic risk score developed using metabolomic profiling of patients with advanced gastric cancer receiving PD‐1 inhibitors plus chemotherapy, based on various metabolites including those enriched in bile acid metabolism pathways, outperformed traditional clinical factors in predicting survival [103]. These findings indicate that the ratios and types of bile acids, especially the balance between primary and secondary or between specific conjugated forms, are important for prediction. For example, the polyphenol castalagin improved anti‐PD‐1 responses in preclinical models by increasing taurine‐conjugated bile acids, revealing a therapeutically targetable metabolic shift [104]. Bile acid profiles can predict outcomes because they are critical signaling molecules that shape the TME. For instance, in lung cancer, secondary bile acids such as DCA promote immune evasion via the TGR5/STAT3/PD‐L1 axis, whereas UDCA and tauroursodeoxycholic acid (TUDCA) often counteract these effects [15, 44]. Therefore, measuring specific bile acid patterns—such as the primary/secondary bile acid ratio or levels of immunomodulatory bile acids like TLCA or UDCA—in readily accessible biofluids is a valuable approach for patient stratification. This approach leverages the functional output of the gut microbiota to predict ICI efficacy, moving beyond taxonomic microbial analysis to a more direct, metabolite‐based biomarker framework [28, 105]. Figure 3 summarizes the bile acid metabolic pathway and its dual effects on antitumor immunity via FXR, TGR5, and RORγt.

## 6 Gut Microbiota Metabolites Influence the TME Checkpoint Expression

### 6.1 Reshaping the Cellular Composition of the TME

Gut microbiota metabolites, upon reaching the tumor site via the circulation, directly and indirectly affect the number and function of tumor‐infiltrating immune cells, fundamentally altering the cellular composition of the TME [106, 107]. This remodeling is a key mechanism by which the microbiome influences antitumor immunity and the efficacy of therapies such as ICIs [108, 109]. For example, SCFAs (butyrate, propionate, acetate) produced by beneficial bacteria during dietary fiber breakdown have been shown to significantly affect immune cell infiltration [110, 111]. Preclinical studies indicate that SCFAs enhance the infiltration of cytotoxic CD8^+^ T cells and effector memory T cells into tumors while simultaneously reducing the presence and suppressive function of Tregs and MDSCs [112]. This change is not limited to cell numbers; SCFAs also improve the metabolic fitness and proliferative potential of T cells, including CAR T cells, which are important for adoptive cell therapies [66, 113]. Other microbial metabolites, such as IPA (a tryptophan derivative), enhance the generation and stemness of progenitor exhausted CD8^+^ T cells (Tpex), a crucial population for maintaining antitumor responses during immunotherapy [36]. Conversely, certain metabolites can create an immunosuppressive environment. The gut microbial metabolite 4‐hydroxybenzeneacetic acid (4‐HPA) promotes CRC progression by activating the JAK2/STAT3 pathway to enhance chemokine (C‐X‐C motif) ligand 3 (CXCL3) expression, which attracts polymorphonuclear myeloid‐derived suppressor cells (PMN‐MDSCs) to the tumor, thereby inhibiting CD8^+^ T‐cell function [47]. This dynamic interaction between metabolites and immune cells underscores the microbiome's role as a systemic regulator of the TME, where the balance between immunostimulatory and immunosuppressive signals dictates the overall antitumor immune response [28, 114].

This profound reshaping of the immune cell landscape alters the cellular sources and expression patterns of PD‐1 and its ligands PD‐L1 and PD‐L2 in the TME, facilitating the transition of a tumor from an “immune‐excluded” or “immune‐desert” phenotype to an “immune‐inflamed” phenotype [115, 116]. In an immune‐inflamed TME, there are abundant PD‐1‐expressing exhausted T cells and PD‐L1‐expressing tumor cells and antigen‐presenting cells, establishing a dominant immune checkpoint axis [117]. Microbial metabolites can modulate this axis by altering the production of these molecules. For example, SCFAs have been shown to enhance PD‐1 expression on tumor‐infiltrating lymphocytes and PD‐L1 on certain cell types, which unexpectedly may increase tumor responsiveness to anti‐PD‐1/PD‐L1 blockade [112, 118]. Patients with more SCFA‐producing bacteria have better outcomes with dual checkpoint blockade (ipilimumab plus nivolumab) in NSCLC, which is linked to increased infiltration of PD‐1^+^ CD8^+^ T cells into the tumor [118]. Also, metabolites of *Lactobacillus johnsonii*, such as IPA and nicotinic acid (NA), can activate pathways like NF‐κB and increase transcription factors such as NR4A2, leading to higher PD‐1 and IFN‐γ production by CD8^+^ T cells, thereby creating a microenvironment more responsive to PD‐1 blockade [119]. Conversely, metabolites that elevate immunosuppressive populations, such as MDSCs or M2‐polarized macrophages, establish an environment with high PD‐L1 levels and T‐cell absence [47, 120]. Kynurenine, a tryptophan metabolite frequently found in tumors, generates an immunosuppressive environment that promotes PD‐L1 expression and T‐cell exhaustion [22, 28]. By altering the proportions and functional states of PD‐1/PD‐L1‐expressing cells—shifting from a landscape dominated by immunosuppressive cells to one filled with engaged, albeit possibly exhausted, cytotoxic T cells—gut microbiota metabolites play a pivotal role in determining whether a TME is permissive or resistant to checkpoint immunotherapy [70]. This metabolic remodeling of the TME establishes a direct link among diet, microbial composition, and the efficacy of therapies targeting the PD‐1/PD‐L1 pathway [114, 121].

### 6.2 Regulating PD‐L1 Expression in Tumor Cells and Stromal Cells

Gut microbiota metabolites can directly modulate the transcription and post‐translational modifications of PD‐L1 in tumor cells by affecting intracellular signaling pathways. For example, sodium butyrate, an SCFA and HDAC inhibitor, enhances PD‐L1 expression in glioma cells by modulating the PI3K/AKT pathway [122]. In contrast, butyrate inhibits IFN‐γ‐induced PD‐L1 upregulation in CRC cells by promoting the acetylation and degradation of STAT1, a key transcription factor for PD‐L1 [123]. This illustrates the context‐dependent and dose‐specific effects of metabolites; high‐dose SP was reported to enhance PD‐L1 expression in CRC cells by stabilizing PD‐L1 mRNA in an m^6^A‐dependent manner via the reader protein IGF2BP3 [19]. In addition to directly affecting tumor cells, microbial metabolites significantly influence the TME by acting on stromal cells. Tumor‐associated macrophages (TAMs) and CAFs are principal targets. SCFAs and other metabolites can modulate immunity; for example, sodium butyrate (NaB) inhibited M2 macrophage polarization and reduced PD‐L1^+^ TAM infiltration in CRC models, an effect dependent on HDAC/TLR4/MyD88 signaling [124]. Moreover, 3‐hydroxybutyrate (3HB), a ketone body produced during a ketogenic diet, was shown to prevent PD‐L1 induction on myeloid cells in the TME, thereby improving the efficacy of PD‐1 blockade [125]. Metabolites also regulate cytokine release from stromal cells, which are important early inducers of PD‐L1. For instance, kynurenine, a tryptophan metabolite produced by IDO1/TDO2 in the TME, creates an immunosuppressive niche that can exhaust T cells and is associated with PD‐L1 expression [126]. In OSCC, the oncometabolite kynurenic acid (KYNA), generated by intratumoral *Streptococcus mutans*, was found to enhance immunosuppressive neutrophils that secrete IL‐1β, thereby contributing to a TME that undermines PD‐L1 blockade therapy [127]. Acetate, another SCFA, can be taken up by tumor cells and increase the transcription of PD‐L1 and other immune evasion genes by acetylating c‐Myc [35]. These complex processes demonstrate that gut microbiota metabolites are important regulators of PD‐L1 expression, acting both directly on tumor cell signaling and indirectly through stromal cell function and cytokine networks, thereby influencing the efficacy of PD‐1/PD‐L1 checkpoint blockade immunotherapy.

## 7 Biomarkers for Predicting ICIs Efficacy Based on Gut Microbiota Metabolites

### 7.1 Metabolite Profile Prediction Models

Non‐targeted or targeted metabolomic analysis of patient serum or feces using techniques such as mass spectrometry is fundamental for identifying distinctive metabolite profiles significantly correlated with clinical responses to ICIs, differentiating complete/partial responses from progressive disease. This approach capitalizes on the profound effects of gut microbiota‐derived metabolites on the TME and therapy outcomes. For example, in BTC patients receiving PD‐1/PD‐L1 inhibitors, liquid chromatography‐tandem mass spectrometry (LC‐MS/MS) analysis identified unique metabolite signatures distinguishing durable clinical benefit (DCB) from NDB cohorts [6]. Certain metabolites, such as some pyrrolidine compounds, were strongly associated with survival outcomes, and when combined with microbial features, enabled the construction of highly accurate predictive models [6]. In NSCLC, baseline plasma metabolomic profiling revealed that tryptophan metabolites, including oxindole‐3‐acetic acid and quinolinic acid, were elevated in patients exhibiting a major pathologic response (MPR) to neoadjuvant anti‐PD‐1 immunochemotherapy, whereas linoleic acid metabolites were linked to non‐response. These distinct metabolic traits, together with specific microbial taxa, were used to build predictive models with area under the curve (AUC) values above 0.85 [128]. Moreover, extensive multi‐omics analyses across pan‐cancer cohorts have integrated fecal microbiome and metabolome data from numerous patients receiving anti‐PD‐1/PD‐L1 therapy to identify cross‐cohort microbial and metabolic signatures indicative of therapeutic response [48]. This integrative approach is important because it captures interactions among different bacteria and their functional effects, making predictions more accurate than taxonomic data alone.

Several specific metabolites show promise as predictive biomarkers, including elevated levels of secondary bile acids (e.g., DCA), certain SCFAs (e.g., butyrate), and indole derivatives (e.g., IPA). The predictive utility of these metabolites is markedly improved when integrated with the abundance of their microbial producers to develop more accurate and mechanistic models. In unresectable HCC, elevated fecal secondary bile acids, specifically UDCA and ursocholic acid, were significantly correlated with the prevalence of *Lachnoclostridium* and linked to an objective response to ICIs [45]. On the other hand, the metabolite phenylacetylglutamine (PAGln) was negatively correlated with ICI response in several cancer types and reduced anti‐PD‐1 efficacy in vivo [48]. Butyrate, an important SCFA, has been shown to be a key immunomodulatory metabolite. In CRC, the enhanced efficacy of PD‐1 antibody therapy by Zhenqi Fuzheng Granule was mediated by elevated gut microbiota‐derived butyrate, which activated the GPR109A pathway to reshape the immunometabolic landscape [129]. In breast cancer models, fucoidan augmented anti‐PD‐1 therapy by altering the gut microbiota and markedly elevating SCFA levels, especially acetic and butyric acids, thereby enhancing effector T‐cell functionality [24]. Other SCFAs have also shown promise; isobutyric acid had the strongest effect among SCFAs in boosting anti‐PD‐1 immunotherapy in cancer models [25]. In addition to SCFAs, tryptophan pathway metabolites are important. In lung cancer, GPs enhanced anti‐PD‐1 therapy by elevating microbial valeric acid and reducing l‐kynurenine, thus improving the kynurenine/tryptophan ratio and inhibiting Tregs [88]. This is similar to findings in CRC, where *Ganoderma lucidum* polysaccharide improved anti‐PD‐1 therapy by lowering the serum kynurenine/tryptophan ratio [130]. Combining metabolite data with microbial signatures—such as the abundance of response‐associated bacteria like *Akkermansia* and *Clostridia_UCG‐014* together with increases in SCFAs—to predict reversal of anti‐PD‐1 resistance creates robust, biology‐based predictive models [131]. These multi‐parameter models, incorporating both microbial community structure and functional metabolic output, represent a powerful approach to identify new therapeutic targets and determine which patients are most likely to benefit from ICI therapy.

### 7.2 Metabolic Function Genes as Surrogate Biomarkers

Metagenomic analysis has become a potent method for functionally characterizing the gut microbiome, transcending taxonomic composition to reveal the prevalence of genes encoding enzymes that facilitate the synthesis or transformation of essential immunomodulatory metabolites. This functional profiling can uncover the genetic capacity for synthesizing metabolites such as SCFAs, secondary bile acids, and tryptophan derivatives, which affect ICI efficacy [28, 132]. For example, genes for bile salt hydrolases (BSHs) and 7α‐dehydroxylase (important for converting primary bile acids to secondary bile acids), or genes in the *but* gene cluster that produce butyrate (e.g., butyrate kinase), can be quantified [133, 134]. This approach links the genetic potential of microbes to the production of bioactive compounds that modulate the immune system both locally and systemically, providing deeper insight than taxonomic signatures alone [135]. The prevalence of these metabolic function genes is strongly associated with better clinical outcomes after ICI therapy. In particular, an increased genetic capacity for butyrate synthesis (evidenced by the *but* gene cluster) and for secondary bile acid production has been correlated with enhanced ICI responses and extended progression‐free survival in multiple cancers, including HCC and melanoma [136, 137]. These genetic signatures functionally complement taxonomy‐based microbial biomarkers, establishing a more direct connection to the immunomodulatory mechanisms influenced by microbial metabolites [48]. For instance, in patients with operable esophageal cancer treated with neoadjuvant chemoradiotherapy and ICIs, a combined analysis of fecal metagenomics and metabolomics identified specific bacterial species and associated metabolic pathways, such as those for primary bile acid and sphingolipid metabolism, that correlated with pathological complete response [138]. Likewise, the gut microbiota's capacity to metabolize one‐carbon compounds, resulting in formate production, has been recognized as a biomarker for enhanced CD8^+^ T‐cell‐mediated antitumor immunity and improved ICI efficacy [137]. Additionally, the metabolite PAGln, produced through specific microbial enzymatic activities, showed a negative correlation with anti‐PD‐1 response in a pan‐cancer analysis, underscoring how functional gene profiles can identify metabolites that directly diminish immunotherapy efficacy [48]. Consequently, profiling metabolic function genes—such as those involved in SCFA synthesis, bile acid transformation, or specific pathways like formate or urocanic acid production—provides a solid, mechanism‐based foundation for developing predictive biomarkers [132, 139]. This functional genomic approach not only predicts response but also identifies microbial metabolic pathways that can be targeted through dietary changes, probiotics, or postbiotic supplementation to reshape the TME and overcome ICI resistance, bringing us closer to precision microbiome‐guided immunotherapy [140, 141].

## 8 Clinical Intervention Strategies Targeting Gut Microbiota Metabolites

### 8.1 Dietary Intervention and Prebiotic/Probiotic Supplementation

Dietary changes, especially those that increase fermentable fiber intake, are key to altering the gut microbiota and enhancing the efficacy of PD‐1/PD‐L1 ICB. High fiber intake enriches fiber‐fermenting bacteria, such as those from the families Ruminococcaceae and *Lachnospiraceae*, leading to increased production of SCFAs (butyrate, propionate, acetate) in the colon and systemically [142]. SCFAs are important immunomodulatory metabolites that can, for example, promote CD8^+^ T‐cell infiltration into the TME and their cancer‐killing activity, a key mechanism for improving anti‐PD‐1/PD‐L1 therapy outcomes [142]. Clinical evidence supports this association: melanoma patients receiving ICIs who regularly consume a high‐fiber diet have significantly better progression‐free survival. This benefit is most pronounced in patients not taking commercial probiotics [143]. Conversely, low‐fiber diets or the use of broad‐spectrum antibiotics, which eliminate beneficial microbial communities, are associated with reduced responses to immunotherapy [143]. The Diet and Immune Effects Trial (DIET) and other ongoing clinical trials are formally testing the hypothesis that a controlled high‐fiber diet can improve the structure and function of the gut microbiome, thereby enhancing systemic and tumor immunity and clinical outcomes in melanoma patients receiving standard‐of‐care ICB [144]. This approach is considered safe and feasible because it uses diet as a modifiable factor to alter the microbial metabolite landscape, particularly SCFAs, to promote an antitumor immune response [145].

The direct administration of specific probiotic strains has emerged as a promising adjunct to immunotherapy, with preclinical models showing that they can overcome resistance to PD‐1/PD‐L1 blockade. In both animal studies and patient cohorts, certain bacterial species—such as *Akkermansia muciniphila*, *Faecalibacterium prausnitzii*, and various *Bifidobacterium* and *Lactobacillus* strains—are consistently associated with better ICI responses [146]. The mechanisms involved are diverse but often involve producing beneficial metabolites or eliminating harmful ones. For instance, the probiotic *Enterococcus* species can release the peptidoglycan hydrolase SagA, which generates immune‐boosting muropeptides that improve antitumor immunity in a NOD2‐dependent manner, thereby enhancing anti‐PD‐L1 therapy efficacy [147]. Similarly, oral administration of *Lactobacillus kefiranofaciens* ZW18 improved gut microbiota composition by increasing the abundance of *Akkermansia* and other beneficial taxa. This was linked to more CD8^+^ T cells in tumors and a better antitumor effect when combined with anti‐PD‐1 treatment in a melanoma model [148]. In addition, preclinical models showed that the probiotic *Bacteroides fragilis* BF839 synergized with anti‐PD‐1 therapy by increasing tumor‐infiltrating CD8^+^ T cells and activating the cGAS‐STING signaling pathway. Retrospective clinical data also suggested that long‐term use of BF839 as an adjuvant was associated with longer overall survival in patients with advanced solid tumors treated with ICIs [149]. These studies indicate that some probiotics can act as “living adjuvants,” modulating both local and systemic gut immunity to facilitate ICI action [150].

Prebiotics are non‐digestible food ingredients that selectively stimulate the growth and/or activity of beneficial gut microorganisms. They are a safe way to alter the host's metabolite profile to support immunotherapy. Inulin, fructooligosaccharides (FOS), galactooligosaccharides (GOS), and other plant‐based polyphenols and fibers are common prebiotics [151]. Prebiotics promote the growth of beneficial bacteria by providing a growth substrate, boost the production of metabolites such as SCFAs and lactate, and enhance the abundance of SCFA‐producing bacteria [151]. For example, the plant polyphenol castalagin, derived from the camu‐camu berry, acts as a prebiotic by boosting *Ruminococcaceae* and *Alistipes* and increasing taurine‐conjugated bile acids; together, these changes reshape the TME and help overcome anti‐PD‐1 resistance in preclinical models [104]. Similarly, bilberry anthocyanin extracts enhance the antitumor effects of anti‐PD‐L1 antibody in mice by increasing the abundance of *Clostridia* and *Lactobacillus johnsonii* in the feces and increasing microbial diversity [152]. Many prebiotics are safe and natural, making them promising candidates for clinical translation. The goal is to establish a microbiota that creates a metabolite environment conducive to antitumor immunity and response to PD‐1/PD‐L1 blockade [151].

### 8.2 Clinical Studies of FMT

FMT is an emerging strategy to modulate responses to ICIs, particularly those targeting the PD‐1/PD‐L1 axis, and may benefit patients with primary or acquired resistance. The basic concept is to transplant fecal microbiota from ICI responders into non‐responders, with the goal of restoring a healthy gut microbial ecosystem. Studies in advanced melanoma first demonstrated that FMT from anti‐PD‐1 responders, followed by re‐challenge with PD‐1 blockade, is safe and can induce clinical responses in some patients with PD‐1‐refractory disease [153]. A key clinical trial showed that this combination was well tolerated and produced clinical benefit in six of 15 patients, accompanied by rapid and durable changes in the microbiota, increased CD8^+^ T‐cell activation, and reduced numbers of interleukin‐8‐expressing myeloid cells in responders [154]. This demonstrated that FMT from responders could alter the TME to circumvent anti‐PD‐1 resistance. In initial trials, no grade 3 adverse events attributable solely to FMT were observed, supporting the safety of this combination in first‐line melanoma treatment [155, 156]. The potential of FMT extends beyond melanoma. A clinical trial involving patients with various advanced solid cancers resistant to anti‐PD‐1 treatment, including gastrointestinal cancers, found that FMT from responders induced durable microbiota changes and clinical benefit in six of 13 patients, with benefits linked to increased cytotoxic T cells and immune cytokines in both blood and tumors [157]. In a phase 2 trial (FMT‐LUMINate) of NSCLC, combining healthy donor FMT with anti‐PD‐1 as first‐line treatment achieved an objective response rate of 80%, meeting the trial's primary endpoint and demonstrating efficacy [158]. Together, these studies show that FMT has the potential to restore ICI efficacy in various cancer types.

FMT works by altering the recipient's gut microbiota composition and associated metabolites, thereby boosting both systemic and intratumoral immunity. After FMT, recipients often show increased abundance of bacterial taxa associated with good ICI responses and decreased abundance of harmful bacteria [156, 157]. A key functional outcome of this microbial remodeling is the alteration of essential immunogenic metabolites. Clinical and preclinical evidence indicates that FMT can increase levels of beneficial microbial metabolites, such as secondary bile acids and SCFAs like butyrate [29, 159]. For example, FMT from donors with a favorable gut mycobiome enterotype sensitized mice to anti‐PD‐1, which was linked to increased butyrate production in vivo [159]. These metabolites directly alter the TME: SCFAs can boost the activity of CD8^+^ T cells and DCs while reducing the activity of immunosuppressive cell types such as MDSCs [70]. Consequently, successful FMT is often associated with measurable improvements in patients’ immune status, including increased intratumoral cytotoxic CD8^+^ T cells, a shift toward a more pro‐inflammatory, antitumor systemic cytokine profile, and decreased T‐cell exhaustion markers [154, 157]. Multi‐omics studies have shown that the gut microbiome remodeled by FMT gives rise to distinct proteomic and metabolomic patterns linked to clinical response [154]. Moreover, introduction of specific beneficial strains, such as *Prevotella merdae* Immunoactis, taken from a responder, has been shown to boost T‐cell activity and slow tumor growth in preclinical models [157]. Therefore, FMT does not simply replace microbes; it also reshapes the host's metabolism and immune system to restore responsiveness to PD‐1/PD‐L1 blockade.

### 8.3 Direct Metabolite Supplementation or Engineered Bacterial Therapy

Direct oral supplementation of specific gut microbiota metabolites, including SCFAs and certain bile acids, constitutes a straightforward approach to modulating the TME and augmenting ICI efficacy. Preclinical studies have shown that oral administration of SCFAs such as butyrate can enhance anti‐PD‐1 therapy. For example, in NSCLC models, butyrate supplementation improved the effectiveness of anti‐PD‐1 immunotherapy by altering TCR signaling in cytotoxic CD8^+^ T cells [17]. Sodium butyrate also augmented the antitumor effects of PD‐1 blockade in a murine glioma model by modifying the TME and elevating levels of antitumor metabolites [122]. In addition to butyrate, other SCFAs such as isobutyric acid have demonstrated potential; oral administration of isobutyric acid significantly augmented the antitumor efficacy of anti‐PD‐1 antibody in carcinoma‐bearing mice [25]. The mechanism often involves epigenetic regulation; butyrate, functioning as an HDAC inhibitor, can increase histone acetylation at promoter regions of genes such as *Pdcd1* and *Cd28*, thereby facilitating PD‐1 and CD28 expression on T cells and enhancing their antitumor activity [17]. Moreover, supplementation with metabolites such as IPA, derived from microbial tryptophan metabolism, has been shown to improve immunotherapy efficacy by influencing T‐cell stemness in pan‐cancer models [36]. However, translating these results into clinical practice is challenging. The ideal human dosing regimen, pharmacokinetics, and reliable delivery methods for these metabolites to achieve consistent and safe systemic or local concentrations remain significant hurdles. Studies have indicated that SCFAs such as acetate and butyrate are elevated in the gut and serum after interventions such as fucoidan supplementation in breast cancer models, but ascertaining the precise oral dosage needed to reproduce these effects in humans without off‐target effects or gastrointestinal discomfort is complex [36]. Furthermore, the bioavailability and stability of these metabolites after oral administration can vary, and their effects may be significantly influenced by the host microbiota and tumor type.

Synthetic biology offers a more advanced and potentially precise solution by creating engineered bacteria intended to colonize the gut or tumor site and continuously produce specific immunomodulatory metabolites. This approach aims to overcome the problems associated with direct supplementation by enabling localized, long‐term production. Scientists are developing engineered bacterial strains, such as attenuated *Salmonella typhimurium* or *Escherichia coli* Nissle 1917, into controllable bioreactors. These bacteria can be programmed with synthetic gene circuits to respond to specific signals in the TME (e.g., low pH, high lactate, hypoxia) and deliver therapeutic payloads. For instance, one study genetically modified *E. coli* Nissle 1917 to convert ammonia—a waste product of tumor metabolism—into l‐arginine, increasing intratumoral l‐arginine levels and improving immunotherapy efficacy [36]. Another approach is to genetically modify bacteria to produce SCFAs directly in the tumor. In addition, scientists have engineered bacteria that release ICIs locally; for example, they engineered *Salmonella* VNP20009 with gene circuits that produce PD‐1 and Tim‐3 single‐chain variable fragments (scFv) inside tumors, blocking immunosuppressive receptors and boosting T cells [160]. An engineered probiotic consortium using a quorum‐sensing system was developed to respond to TME factors (pH, hypoxia, lactate) and coordinate the release of a PD‐L1 nanobody and lactate‐depleting enzymes, demonstrating substantial tumor suppression in CRC models [161]. Another innovative idea is to genetically modify bacteria to produce immune‐modulating metabolites; for example, engineered *E. coli* Nissle 1917 was used to deliver an immunotoxin (αPD‐L1‐PE38) directly to tumor tissues [162]. Bacteria have also been engineered to produce cytokines such as IL‐15 and IL‐15Rα in a thermally responsive manner following microwave ablation therapy, thereby inducing antitumor immunity [163]. Researchers have investigated the use of outer membrane vesicles (OMVs) from genetically modified Gram‐negative bacteria that display the PD‐1 ectodomain, combining immune activation with checkpoint inhibition [164]. These engineered bacterial therapies show great promise; however, most are still in preclinical development. Major challenges for clinical translation include ensuring the safety and controllability of these live biotherapeutic products, preventing systemic dissemination or unintended immune activation, ensuring robust and stable colonization of the human gastrointestinal tract or tumor, and navigating complex regulatory pathways. Future research should focus on enhancing the precision of these systems, employing ultrasound‐driven [164] or thermal‐responsive [163] gene expression controls, and conducting extensive toxicology studies to facilitate clinical trials.

## 9 Challenges and Limitations in Clinical Translation

### 9.1 Complexity and Contradiction of Metabolite Actions

The complex and often opposing functions of gut microbiota‐derived metabolites in modulating the PD‐1/PD‐L1 axis pose a considerable obstacle to their use as therapeutic targets or predictive biomarkers. A prime example is AhR ligands, such as microbial tryptophan catabolites, whose effects are highly context‐dependent. For instance, the microbial metabolite I3C can protect against ICI‐induced colitis by activating the host AhR/IL‐22 pathway and reshaping the gut microbiota toward a butyrate‐producing profile, thereby reducing toxicity without compromising antitumor efficacy [37]. On the other hand, kynurenine and other tryptophan‐derived metabolites are usually linked to immunosuppression. In NSCLC, elevated IDO1 expression, which signifies active tryptophan catabolism, is paradoxically associated with an enhanced response to PD‐1/PD‐L1 blockade, likely because it reflects an inflamed, immune‐active TME rather than acting as a direct contributor to resistance [165]. This duality also applies to concentration‐dependent effects. In CRC, low‐dose SP (10 mM) inhibits cell proliferation, whereas a high dose (40 mM) enhances PD‐L1 expression via the IGF2BP3 axis, potentially facilitating immune evasion while simultaneously creating a targetable vulnerability for combination therapy with PD‐1/PD‐L1 blockade [19]. Butyrate, another SCFA, also has antitumor effects through HDAC inhibition. It can mitigate PD‐1/PD‐L1 inhibitor‐induced cardiotoxicity by polarizing colonic macrophages away from an M1‐like phenotype via the PPARα‐CYP4X1 axis [166]. Conversely, butyrate has been shown to enhance PD‐L1 expression in glioma cells through the PI3K/AKT pathway, potentially facilitating immune evasion [122]. These opposing effects demonstrate that a metabolite's action is not intrinsic but is instead influenced by tumor type, local immune cell composition, metabolite concentration, and the broader metabolic network. The “metabolite‐metabolite” interactome is even more complex because its components are interconnected. Microbial communities do not produce metabolites in isolation; they operate within a dynamic network where one metabolite can affect the production or function of another. For instance, the efficacy of PD‐1/PD‐L1 therapy can be influenced by a consortium of metabolites, including SCFAs (butyrate, acetate), tryptophan derivatives, inosine, and secondary bile acids, which collectively regulate immune cell differentiation, antigen presentation, and checkpoint expression [15, 28]. Focusing on the effects of a single metabolite such as butyrate may not accurately reflect in vivo biology, as its synthesis by bacteria such as *Prevotellaceae* is associated with mitigation of PD‐1/PD‐L1 inhibitor‐induced cardiotoxicity via the PPARα‐CYP4X1 pathway in colonic macrophages [166], an effect that relies on a functional microbial ecosystem. Therefore, to determine how microbiota metabolites can be used in medicine, we need to move beyond reductionist approaches and adopt systems‐level analyses that consider their network interactions, contextual dependencies, and bidirectional communication along the gut‐tumor axis [15, 28].

### 9.2 Individual Differences and Standardization Challenges

Host‐specific factors significantly influence the composition of the gut microbiota and its metabolic output, making it difficult to develop universal therapeutic strategies targeting the PD‐1/PD‐L1 axis. An individual's genetic background, baseline diet, concomitant medications, and tumor type all interact to create a unique microbial ecosystem and metabolite profile, which in turn determines the response to ICIs [11, 167]. For example, in HCC, ICI efficacy is strongly linked to distinct fecal microbial signatures and bile acid profiles; responders had higher levels of *Lachnoclostridium* and UDCA, whereas non‐responders had higher levels of *Prevotella 9*, demonstrating tumor‐type‐specific microbiota interactions [45]. Long‐term survivors of lung cancer on anti‐PD‐1/PD‐L1 therapy also have a co‐evolved, compact microbial community that produces immunomodulatory SCFAs such as butyrate and propionate; however, this metabolic signature is not present in all patients [168]. This person‐to‐person variability also applies to the risk of immune‐related adverse events (irAEs). For example, baseline gut microbiota composition is a key factor in conditions such as colitis, and certain microbial sequences can mimic autoimmune antigens, causing adverse immune reactions in some patients [168, 169]. The gut‐liver axis exemplifies the complexity further: the interaction between gut microbial metabolites and liver immune cells varies significantly, affecting ICI responses in liver cancers in a manner dependent on the etiology of liver disease, such as metabolic‐associated fatty liver disease (MAFLD) [170, 171]. Because of this inherent biological diversity, the search for a universally effective microbial‐ or metabolite‐based adjuvant therapy is challenging, underscoring the need to move toward personalized approaches that consider each individual's unique microbial and metabolic landscape [108].

The lack of standardization in the preparation and administration of live biotherapeutic products, including FMT and probiotics, exacerbates individual variation by introducing considerable batch‐to‐batch variability and uncertainty regarding their efficacy and safety. The process for selecting donors for FMT is not consistent, and what constitutes a “healthy” or “therapeutic” donor microbiota is not clearly defined, leading to variable clinical outcomes [169, 172]. Production methods for these products, such as stool processing, bacterial growth, and final product manufacturing, differ significantly, affecting the viability, diversity, and function of the microorganisms [173]. This is important because specific bacterial strains, not just broad taxonomic groups, are involved in therapeutic effects. Studies have identified strain‐level differences within species such as *Faecalibacterium prausnitzii* and *Escherichia coli* that are linked to clinical phenotypes in ICI therapy [168]. Moreover, optimal dosing schedules, administration routes, and timing relative to ICI treatment are based on empirical evidence and have not been validated [174]. The therapeutic potential of FMT in addressing ICI resistance or managing refractory irAEs is tempered by practical challenges, as the transfer of a complex, undefined microbial community poses risks of pathogen transmission or unexpected immunological alterations. Likewise, although specific probiotics or defined microbial consortia offer a more regulated option, their effects may vary based on context and the recipient's pre‐existing microbiota, possibly leading to diverse outcomes [175, 176]. The field urgently needs strict, universally accepted guidelines for donor screening, product characterization (including metagenomic and metabolomic profiling), potency testing, and clinical trial protocols to ensure that microbiota‐based treatments are safe, effective, and reproducible [10, 177].

## 10 Future Research Directions and Prospects

### 10.1 In‐Depth Mechanism Exploration and Multi‐Omics Integration

We do not fully understand how specific gut microbiota metabolites regulate PD‐1/PD‐L1 expression and function in different cell types within the TME. To better understand these complex interactions, future research should employ high‐resolution technologies. Combining single‐cell RNA sequencing (scRNA‐seq) with spatial metabolomics is a powerful new approach. While scRNA‐seq can reveal the transcriptional profiles and PD‐1/PD‐L1 status of various immune and tumor cell populations in a heterogeneous TME, spatial metabolomics techniques such as mass spectrometry imaging (MSI) can localize specific microbial metabolites within tumor tissue [178]. This combined approach can determine whether immunosuppressive metabolites like kynurenine accumulate in certain regions, such as areas rich in Tregs or TAMs, and how these “metabolite hotspots” are linked to localized upregulation of PD‐1 on exhausted CD8^+^ T cells or PD‐L1 on neighboring cells [126]. Studies have demonstrated that the tryptophan‐kynurenine‐AhR pathway generates immunometabolic microdomains that inhibit T‐cell function and enhance PD‐1 expression, a phenomenon that can be spatially elucidated using these technologies [40, 126]. In addition, methods such as GeoMx Digital Spatial Profiling allow simultaneous examination of protein expression (e.g., PD‐L1) and metabolic enzyme activity in morphologically defined regions of interest, connecting the presence of metabolites to checkpoint protein expression in specific cell types [178]. This kind of high‐resolution mapping is very important. For example, high‐dose SP has been shown to increase PD‐L1 expression in CRC cells via the IGF2BP3 axis, an effect that likely varies by tumor and cell type [19]. Using these integrated tools, researchers can move beyond correlative associations to delineate causal, spatially defined relationships between microbial metabolites and the PD‐1/PD‐L1 axis, ultimately pinpointing specific cellular targets for therapeutic intervention.

To apply mechanistic insights in clinical practice, large, long‐term cohort studies that systematically combine multi‐omics data are needed. These studies should recruit patients undergoing PD‐1/PD‐L1 blockade across diverse cancer types and systematically collect biospecimens (stool, blood, tumor tissue) along with comprehensive clinical metadata over time. Combining metagenomics (to profile gut microbial composition and functional potential), metabolomics (to quantify circulating and fecal metabolites), immunophenotyping (to assess peripheral and intratumoral immune cell subsets), and clinical outcomes (response, survival, toxicity) will create a dataset of unprecedented scale [6, 7]. For instance, a prospective study on BTC successfully used metagenomics and LC‐MS/MS metabolomics to identify specific bacteria and metabolites (e.g., *Alistipes* and pyrrolidine) linked to durable clinical benefit from immunotherapy and created highly accurate predictive models [6]. Similarly, combining 16S rRNA sequencing with untargeted metabolomics in patients with extensive‐stage small cell lung cancer showed that beneficial bacteria such as *Faecalibacterium* increased after treatment and that SCFAs increased in responders, with distinct metabolites enriched in PD‐1 checkpoint pathways [7]. Because of the large volume and complexity of these data, artificial intelligence (AI) and machine learning (ML) algorithms are needed to find non‐linear patterns and make robust predictions. For example, integrative analysis can reveal causal chains. Machine learning methods applied to multi‐omics data from patients with CRC have identified links between specific gut genera, immunomodulatory metabolites, and tumor immunity gene signatures, suggesting a systems‐level understanding of how the microbiota affects treatment efficacy [179]. The ultimate goal is to create clinically applicable AI tools that personalize treatment by using baseline or early‐treatment multi‐omics profiles to identify patients who might benefit from microbiota‐modulating interventions (e.g., probiotics, prebiotics, FMT), thereby making tumors more sensitive to checkpoint blockade and moving us closer to truly precision immuno‐oncology [180, 181].

### 10.2 Development of Next‐Generation Precision Microbial Therapies

Microbial‐based interventions for improving PD‐1/PD‐L1 immunotherapy are moving from broad‐spectrum methods such as FMT to more specific and controllable methods such as defined microbial consortia and engineered live biotherapeutic products (LBPs). FMT, which transfers the entire gut microbial community from a healthy donor to a patient, has shown clinical promise for overcoming ICI resistance and treating ICI‐related colitis [182, 183]. However, its use is limited by donor variability, potential safety risks, and the difficulty of standardizing a complete microbial ecosystem [180, 184]. This has led to the development of “defined microbial consortia,” which are carefully selected mixtures of specific bacterial strains known to have therapeutic effects. For example, clinical and preclinical studies have identified important beneficial taxa—*Akkermansia muciniphila*, *Bifidobacterium* spp., *Faecalibacterium prausnitzii*—associated with better ICI responses [185]. Administering a defined mix of these strains is safer, more consistent, and clearer than FMT. The cutting edge in this field is the development of “engineered bacteria” (LBPs) based on synthetic biology. These are genetically modified probiotic strains designed to perform specific therapeutic functions in the gut or TME. For instance, scientists have engineered *E. coli* Nissle 1917 to produce and release anti‐PD‐1 nanobodies or immunomodulatory cytokines such as CXCL13 and IL‐12 in tumors [186, 187]. This approach aims to achieve stable colonization, precise spatial and temporal control of drug delivery, and sustained production of target metabolites or therapeutic proteins, thereby maximizing antitumor immunity while minimizing systemic toxicity. The goal is to develop LBPs that are safe, controllable, and capable of maintaining the desired immunomodulatory effects to synergize with and improve PD‐1/PD‐L1 blockade therapy [29].

With the growth of live biotherapeutics, research is also focused on circumventing the challenges associated with administering live bacteria by directly targeting the molecular effectors of microbiota‐immune crosstalk. This involves identifying small‐molecule agonists or antagonists of specific microbial metabolite receptors or designing metabolite analogs that can be used as drugs in combination with ICIs. For example, kynurenine activates AhR on immune cells, which can render the TME less immune‐friendly and increase PD‐L1 levels, thereby increasing the likelihood of ICI resistance [20, 126]. Researchers are investigating small‐molecule AhR antagonists to block this pathway and potentially reverse immunosuppression, making tumors more sensitive to PD‐1/PD‐L1 blockade [21]. On the other hand, butyrate and other SCFAs signal through GPR43/GPR41 and inhibit HDAC, helping T cells fight tumors and maintain stemness [18, 122]. Thus, developing stable SCFA analogs or GPR43/41 agonists could enhance ICI efficacy without the challenges associated with bacterial colonization. Similarly, modulating bile acid metabolism with FXR agonists or antagonists is another strategy for fine‐tuning immune responses [15]. Using metabolite analogs or receptor‐targeting small molecules is a pharmacological approach that aims to improve pharmacokinetic and safety profiles compared to live bacteria. This facilitates systemic administration of the appropriate dose, potentially avoiding issues related to variable bacterial engraftment, pathogenicity risks, and the complex regulations associated with live biotherapeutic products. By examining these downstream molecular mechanisms, researchers aim to develop off‐the‐shelf, precision drugs that can be combined with ICIs to modify the immune system in a controlled and effective manner [11, 188].

### 10.3 Optimizing Clinical Trial Design and Endpoints

Translating our knowledge of gut microbiota metabolites and PD‐1/PD‐L1 checkpoint regulation into useful treatments requires more rationally designed clinical trials. Incorporating rigorous, longitudinal monitoring of both gut microbiota composition and circulating metabolite profiles into trial protocols is a crucial step [28]. Adaptive trial designs are valuable in this emerging field because they allow researchers to identify patient subgroups most likely to benefit from specific microbiome‐modulating strategies based on baseline microbial or metabolic signatures [132]. For example, patients with higher levels of certain beneficial taxa or metabolites (e.g., SCFAs, inosine) may respond better to ICI therapy when combined with a microbiome‐targeted intervention [46, 145]. Conversely, adaptive designs can also help identify patients at risk for primary resistance, which may be associated with dysbiotic signatures or the presence of immunosuppressive metabolites such as kynurenine [188]. By integrating these complex biological data points in real time, trials can move away from a one‐size‐fits‐all approach and adopt more precise patient stratification and rapid evaluation of combination therapies tailored to each individual's microbial and metabolic landscape [181].

## 11 Conclusion

Research on gut microbiota‐derived metabolites has identified the microbiome as a crucial factor influencing the efficacy of cancer immunotherapy. As this review has shown, these metabolites are important molecular messengers that control the immune system, reshape the TME, and affect the clinical efficacy of ICIs. The field is moving from establishing correlations to dissecting mechanisms and translating them into treatments. The biggest challenge is to harness this complexity for patient benefit.

SCFAs, tryptophan derivatives, and bile acids are three major classes of metabolites that interact with the PD‐1/PD‐L1 pathway through distinct but interconnected mechanisms, including epigenetic regulation, receptor signaling, and metabolic reprogramming. This wide‐ranging effect indicates that the gut microbiome influences ICIs through a complex, interconnected regulatory system. It is important to understand that these metabolites can have context‐dependent and sometimes opposing effects; for example, butyrate exerts different effects on Treg versus cytotoxic T‐cell function.

Translating this science into biomarkers and treatments shows both great promise and significant challenges. Metabolomic signatures and functional metagenomics show promise for predicting ICI response. Interventional strategies such as dietary changes, FMT, and metabolite supplementation offer new ways to overcome resistance. However, inter‐individual differences in microbiota composition and metabolic output make it difficult to use universal biomarkers or therapies that work for everyone. Moreover, the transition from preclinical models to standardized clinical practice is still fraught with issues regarding manufacturing, donor selection, safety, and trial design.

Future progress requires concerted efforts on multiple fronts. Mechanistically, combining multi‐omics technologies with well‐designed longitudinal cohorts will help the field move from association to causal inference. Next‐generation precision microbial medicines, such as engineered probiotics or synthetic consortia, must be guided by trials that prioritize patient safety, mechanistic endpoints, and microbial baseline stratification. By fostering interdisciplinary collaboration, the promise of modulating microbial metabolites to improve PD‐1/PD‐L1 blockade can become a reality, improving the lives of cancer patients worldwide.

## Author Contributions

**Xuanyou Fang:** methodology. **Shuai Yuan:** data curation. **Weijian Mai:** validation and project administration. **Xuanyou Fang** and **Shuai Yuan:** writing – original draft. **Xuanyou Fang** and **Shuai Yuan:** writing – review and editing.

## Funding

The authors have nothing to report.

## Ethics Statement

This article is a review paper and does not contain any studies with human participants or animals performed by the authors.

## Conflicts of Interest

The authors declare no conflicts of interest.

## Data Availability

Data sharing is not applicable to this article as no new data were created or analyzed in this study.
